# A new approach for understanding the association between chronic stress in childhood and psychotic symptoms in schizophrenia patients from mediating role of lncRNA

**DOI:** 10.1038/s41537-026-00755-w

**Published:** 2026-04-24

**Authors:** Zhiyong Mi, Yan Yan, Lingming Kong

**Affiliations:** 1 Psychiatry Department, The 5th People’s Hospital of Luoyang, Luoyang, Henan China; 2 Psychiatry Department, Mental Health Center of Qingpu district, Shanghai, China; 3 Psychiatric Research Center, No. 904th Hospital of Joint Logistic Support Force, Changzhou, Jiangsu China

**Keywords:** Epigenetics in the nervous system, Psychiatric disorders, Schizophrenia

## Abstract

Schizophrenia (SCZ) remains an etiological and therapeutic challenge, this article aims to explore the mediating role of lncRNA between chronic childhood stress and SCZ for providing a scientific basis of SCZ prevention. 200 SCZ patients and 200 healthy controls (HCs) were enrolled by convenience sampling. Positive and Negative Syndrome Scale (PANSS) and Childhood Chronic Stress Questionaire (CCSQ) were employed for mental assessment. lncRNA and cortisol level in peripheral blood were respectively detected by real-time quantitative reverse transcription PCR and enzyme-linked immunosorbent assay. The results showed scores of CCSQ, peer bullying (PB), abuse and neglect (AN), adverse life events (ALE) and cortisol were higher, while ΔCT values of NONHSAT089447 and NONHSAT021545 were lowered in SCZ patients (*P* < 0.05 or 0.01). Area under the curve of combined receiver operating characteristic curve for NONHSAT089447, NONHSAT021545, NONHSAT041499 was 0.814 with sensitivity and specificity of being 0.650, 0.880, respectively. Correlation analysis suggested scores of CCSQ, PB, AN, ALE as well as cortisol positively and ΔCT values of NONHSAT089447, NONHSAT021545 negatively correlated with scores of PANSS, positive symptom subscale (PSS), negative symptom subscale (NSS), general pathological symptom subscale (GPSS) in SCZ group (*P* < 0.05 or 0.01). NONHSAT089447 and NONHSAT021545 also played partial mediating roles between chronic childhood stress and psychotic symptoms with accounting proportions of 54.83% and 41.53% for total effect, respectively (*P* < 0.001). Taken together, we established a new model for SCZ in which exposure to chronic childhood stress could induce pathological process via mediating effects of NONHSAT089447 and NONHSAT021545.

## Introduction

Schizophrenia (SCZ) as a mental condition characterized with obvious disturbances including positive symptoms (eg, hallucinations, delusions), negative symptoms (eg, avolition, anhedonia), cognitive impairment, and psychosocial-function decline, remained an etiological and therapeutic challenge^1,2^. One of the basic consensuses about SCZ was that bidirectional relationship of genetic susceptibility and environmental stimuli may play a vital role in pathogenesis process, the neurodevelopmental hypothesis proposed genetic risk of SCZ referring to as a neurodevelopmental disorder was likely dynamic and context dependent with effects of genetic risk varying spatiotemporally during neurodevelopmental continuum. It was believed a variety of environmental factors may interact with genetic risk factors during pre- or postnatal period and/or during adolescence such as interpersonal violence and abuse of physics, emotion, sex, may induce disturbances of macro- and micro-connectivity in brain regions involving the prefrontal, temporal and parietal cortices, hippocampus, as well as a reduction in gray and white matter volume, a disturbed synaptic plasticity, loss of oligodendrocytes, impaired myelination^3–6^, these pathophysiological changes may ultimately induced SCZ onset.

SCZ is a multifactorial disease with a series of risk factors, abnormalities of cytokine, hormone, neurotransmitter, and psychosocial function relating to stress from the internal and external environment are considered to be associated with SCZ. Dysregulation in the systems involved in the stress response was a crucial component of SCZ pathophysiology. Individuals at risk of developing SCZ usually exhibited hyperreactivity to stress and impairment in cognitive performance, both of which are referred to as SCZ vulnerability markers, nevertheless, transcranial direct current stimulation (tDCS) as a non-invasive neurostimulation method for improving psychotic symptoms in SCZ patients could reduce cortisol release in response to stress and prevented stress-induced impairment in reality monitoring^7^. Further research showed chronic childhood trauma was one of the key determinants in shaping the psychological and physiological susceptibilities to stress in SCZ patients, insufficient mentalizing ability and fragmented sense of self as disease susceptibilities in SCZ patients which were associated with psychopathological process may lead to disability in emotion regulation and social contact for their difficulties in integrating perceptions, memories, thoughts, and emotions related to themselves and others. Furthermore, attachment insecurity and alexithymia were confirmed as the mediating variables between childhood trauma and low mentalizing ability in SCZ patients, that is, childhood trauma could impact mentalizing ability development by shaping maladaptive personality traits (eg, insecure attachment and alexithymia)^8–10^. Early life stress (ELS) could seriously damage the brains’ normal growth and development with long-term psychological and physiological repercussions in neurological and behavioral changes, some studies reported ELS impaired memory, emotion regulation, motivation by reducing hippocampal volume, sensitizing the stress response of amygdala and altering neurotransmitter system function, such as serotonin and dopamine. Additionally, ELS disrupted the general development process of prefrontal cortex, leading to deficits in decision-making, planning, and impulse control^11–14^. These psychological and physiological dysfunctions associated with ELS may eventually induce the SCZ pathological process and contribute to the genesis of psychotic symptoms in SCZ patients.

Srivastava et al. argued previous studies in both pre-genomic and post-genomic era, however, have failed to fully elucidate genetic basis for SCZ. In recent years, the epigenetic hypothesis of SCZ—the process which contributed to changes in gene expression without altering DNA sequence—may help to better understand the mechanisms leading to SCZ pathogenesis, the current researches mainly focused on mediating mechanisms or bridges of the interaction between environmental risk factors and genetic susceptibility including DNA methylation, histone modification, non-coding RNA^15^. As an actively evolving family of non-coding RNAs, long non-coding RNA (lncRNA) comprised a group of single-stranded RNA with lengths longer than 200 nucleotides, has been confirmed to exert a regulatory role in SCZ diagnosis and treatment. One previous study found expression levels of lncRNAs(NONHSAT089447, NONHSAT021545, NONHSAT041499) in the peripheral blood of SCZ patients were upregulated, whereas NONHSAT089447 and NONHSAT041499 were downregulated after antipsychotic drug treatment, lncRNA expression alteration before- and after-treatment was observed to be associated with improvement of psychotic symptoms^16^. Despite their limited protein-coding potential, lncRNAs were known for their great importance in achieving global transcriptomic regulation in a spatiotemporal expression profile, especially in complex tissue like the brain^17^, thereby, aberrant lncRNA expression profile may impair brain development course which may ultimately induce the pathological process of SCZ. On the other hand, Lux in one review for epigenetic programming effects of early life stress proposed a dual-activation hypothesis that combined activation of stress-related neural networks and stressor-specific sensory networks led to both neural and hormonal priming of epigenetic machinery, which sensitizes these networks for developmental programming effects. This may furtherly generate functional mal-adaptations associated with neuropsychiatric disorders^18^. Hence, specific expression profile of lncRNAs (NONHSAT089447, NONHSAT021545, NONHSAT041499), as one key epigenetic modifier, may mediated the association between ELS and psychotic symptoms in SCZ patients. Nevertheless, to the best of our knowledge, there is still no empirical evidence for the hypothesis until now.

In summary, chronic childhood stress, exaggerated stress response, aberrantly expressed lncRNA may be risk factors for SCZ and lncRNA plays a regulatory role between chronic stress in childhood and psychopathological symptoms in SCZ patients. This study proposes the following hypotheses: (1) Chronic childhood stress, cortisol, and lncRNA have predictive effects on psychotic symptoms in SCZ patients; (2) lncRNAs (NONHSAT089447, NONHSAT021545, NONHSAT041499) are mediators between chronic stress in childhood and psychotic symptoms in SCZ patients.

## Results

### Demographic characteristics of study participants

There was no significant difference in age (*t* = 1.315, *P* = 0.189), sex ratio (*χ*2 = 1.447, *P* = 0.229) between groups (Table 1).

**Table 1:** Demographic characteristics of SCZ patients and healthy controls.

Variable	SCZ group	Control group	*t* or *χ*^2^	*P*
Age(years)	33.34 ± 6.09	32.56 ± 5.77	1.315	0.189
Sex(male/female)	99/101	87/113	1.447	0.229

### Between-group comparison for chronic stress in childhood and cortisol level

The independent sample *t* test showed the scores of CCSQ, PB, AN, ALE and cortisol level in SCZ patients were significantly higher than the healthy controls (*P* < 0.05 or 0.01, Table 2).

**Table 2:** Comparison for chronic stress in childhood and cortisol level in SCZ patients and control group ($\overline{x}$±SD**)**.

Dimension	SCZ group	Control group	*t*	*P*	*Cohen’s d*
PB	30.43 ± 17.84	24.56 ± 15.37	3.522	<0.001	0.348
AN	57.05 ± 36.23	48.64 ± 34.03	2.393	0.017	0.238
ALE	30.11 ± 19.07	25.30 ± 16.44	2.699	0.007	0.268
CCSQ	117.58 ± 70.13	98.50 ± 62.55	2.871	0.004	0.285
cortisol	47.51 ± 32.39	22.92 ± 15.90	9.636	<0.001	0.869

### Comparison of lncRNA between SCZ patients and control group

The Mann-Whitney U-test showed that ΔCT values of NONHSAT089447, NONHSAT021545 except NONHSAT041499 were significantly decreased in SCZ group than the control group (*P* < 0.05 or 0.01, Table 3).

**Table 3:** Mann-Whitney U-test for expression level of lncRNA in SCZ patients and control group[ΔCT median, M(Q1, Q3)].

Seq name	SCZ group	Control group	*Z*	*P*
NONHSAT089447	3.106(1.023–7.923)	6.143(0.644–10.103)	–2.746	0.006
NONHSAT021545	3.609(1.253–8.359)	5.489(1.060–10.249)	–2.251	0.024
NONHSAT041499	4.948(2.132–8.706)	6.099(0.663–10.145)	–1.547	0.122

### ROC curve for discriminatory value of lncRNA on the SCZ severity

**Fig. 1:**
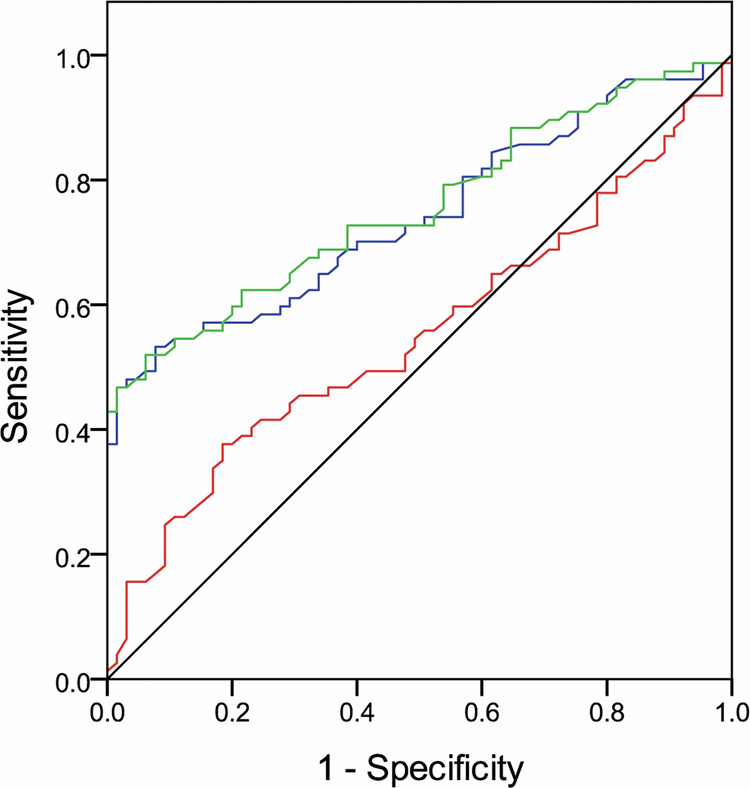
ROC curve for differentiating value of lncRNAs expression on SCZ severity. The blue, green, and red curve indicates NONHSAT089447, NONHSAT021545, NONHSAT041499, respectively.

**Fig. 2:**
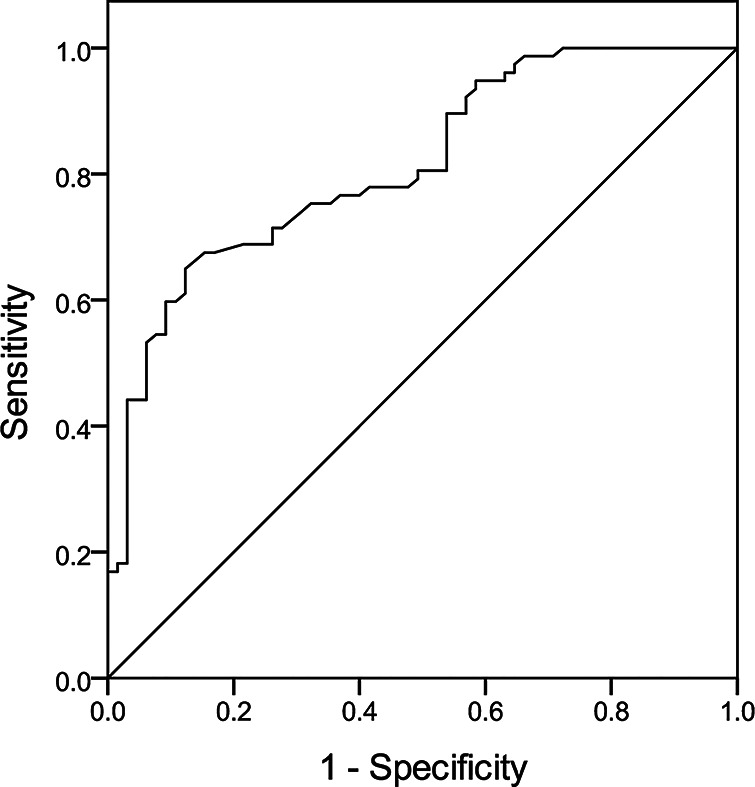
Combined ROC curve of aberrant expressed lncRNAs for differentiating SCZ severity.

According to the test results of the Chinese cooperative research group for PANSS in clinical practice, the cut-off values of raw PANSS score at the 25th, 50th, and 75th percentiles were 73, 81, and 92^21^. The patients with raw PANSS score equal to or less than 73 were assigned into the mild group and equal to or higher than 92 severe group. ROC curve was constructed using patient grouping as state variable and lncRNAs (NONHSAT089447, NONHSAT021545, NONHSAT041499) as test variables. The results showed NONHSAT089447, NONHSAT021545 except NONHSAT041499 significantly differentiating the SCZ severity with area under the curves (AUCs) of being 0.740, 0.755(Fig. 1 and Table 4). The AUC of combined ROC curve of these three lncRNAs was 0.814 with sensitivity and specificity of being 0.650, 0.880, respectively (Fig. 2), Youden index with a maximum value of 0.526 was optimal cut-off threshold of SCZ severity.

**Table 4:** Results of ROC curve for differentiating value of lncRNA on SCZ severity.

Test variables	AUC	*P*	Asymptotic 95% confidence interval		Sensitivity	Specificity
			Lower bound	Upper bound		
NONHSAT089447	0.740	<0.001	0.660	0.821	0.530	0.910
NONHSAT021545	0.755	<0.001	0.676	0.833	0.520	0.940
NONHSAT041499	0.551	0.298	0.456	0.646	0.380	0.810
Combined ROC curve	0.814	<0.001	0.745	0.883	0.650	0.880

### Correlation analysis for chronic childhood stress, cortisol, lncRNA and psychotic symptoms in SCZ group

Pearson’s correlation was employed for association analysis of psychotic symptoms and chronic childhood stress, cortisol and Spearman’s correlation was used for lncRNA expression level and psychotic symptoms. The correlation analysis showed that scores of CCSQ, PB, AN, ALE as well as cortisol positively and ΔCT values of NONHSAT089447, NONHSAT021545 negatively correlated with scores of PSS, NSS, GASS, PANSS in SCZ group (*P* < 0.01, Table 5).

**Table 5:** Correlation analysis for chronic stress in childhood, cortisol, lncRNA, and psychotic symptoms in SCZ patients(*r*).

Factor	PB	AN	ALE	CCSQ	cortisol	NONHSAT089447	NONHSAT021545	NONHSAT041499
PSS	0.360^**^	0.377^**^	0.337^**^	0.383^**^	0.316^**^	–0.255^**^	–0.264^**^	0.002
NSS	0.456^**^	0.505^**^	0.433^**^	0.475^**^	0.302^**^	–0.268^**^	–0.283^**^	–0.023
GPSS	0.497^**^	0.550^**^	0.490^**^	0.532^**^	0.427^**^	–0.373^**^	–0.387^**^	–0.060
PANSS	0.607^**^	0.652^**^	0.573^**^	0.637^**^	0.446^**^	–0.396^**^	–0.414^**^	–0.057

**P < 0.01.

### Mediating-effect analysis for lncRNA between chronic childhood stress and psychotic symptoms in SCZ Group

According to the mediating-effect test procedure^22^, Hierarchical regression analysis was conducted in the following three steps: In the first step, PANSS was used as dependent variable and CCSQ as independent variable, the statistical analysis showed that CCSQ had a significant predictive effect on PANSS (*P* < 0.001); In the second step, NONHSAT089447 and NONHSAT021545 were taken as dependent variables respectively, and CCSQ as the independent variable, it was found that CCSQ had a significant predictive effect on ΔCT values of NONHSAT089447 and NONHSAT021545 (*P* < 0.001); In the third step, PANSS was taken as the dependent variable, and CCSQ, NONHSAT089447 or CCSQ, NONHSAT021545 was taken as independent variables. The results showed that CCSQ, NONHSAT089447, NONHSAT021545 were still significantly associated with PANSS (*P* < 0.001). From the direct effect to the mediating model, the predictive effect of chronic stress in childhood on SCZ remained significant, while the path coefficients were decreased, indicating that NONHSAT089447 and NONHSAT021545 played partial mediating roles between chronic childhood stress and psychotic symptoms. The mediating effects of NONHSAT089447 (Table 6) and NONHSAT021545 (Table 7) accounted for 54.83% (0.945*0.123/0.212) and 41.53% (0.710*0.124/0.212) of the total effects, respectively.

**Table 6:** Hierarchical regression analysis for mediating effect of NONHSAT089447 between chronic stress in childhood and psychotic symptom in SCZ patients.

Steps	DV	IV	Beta	Standard error	t	*P*
Step1	PANSS	CCSQ	0.212	0.019	11.164	<0.001
Step2	NONHSAT089447	CCSQ	–0.123	0.005	–4.493	<0.001
Step3	PANSS	CCSQ	0.190	0.019	9.838	<0.001
		NONHSAT089447	–0.945	0.251	–3.765	<0.001

**Table 7:** Hierarchical regression analysis for mediating effect of NONHSAT021545 between chronic stress in childhood and psychotic symptom in SCZ patients.

Steps	DV	IV	Beta	Standard error	t	*P*
Step1	PANSS	CCSQ	0.212	0.019	11.164	<0.001
Step2	NONHSAT021545	CCSQ	–0.124	0.005	–4.711	<0.001
Step3	PANSS	CCSQ	0.188	0.019	9.718	<0.001
		NONHSAT021545	–0.710	0.257	–3.934	<0.001

## Materials and methods

### Participants

#### Study group

From January 2022 to December 2024, 200 SCZ patients including 99 males and 101 females aged 20–42 (33.34 ± 6.08) years old were continuously enrolled by convenient sampling. All participants were first-episode cases diagnosed as SCZ according to Diagnostic and Statistical Manual of Mental Disorders, Fifth Edition (DSM-V) without histories of blood transfusion and severe trauma or infection within 3 months^19^. All SCZ patients were not taking any antipsychotic medications before study enrollment. The patients with histories of craniocerebral injury, hyperthyroidism, diabetes, abuse of psychoactive substances or other drugs, serious life event experiences within the past six months, such as divorce, unemployment, traffic accidents, natural disasters, and pregnant or lactating patients were excluded.

#### Control group

From January 2022 to December 2024, 200 healthy volunteers, 87 males and 113 females aged 17–42 (32.56 ± 5.77), were also enrolled by convenient sampling. Inclusion criteria: All the healthy volunteers in control group with no disease or family histories of severe mental disorders such as schizophrenia, bipolar disorder, panic disorder, major depressive disorder, personality disorder and blood transfusion history in the past 3 months. The participants with histories of infection, major life event experiences in the past 6 months, psychoactive substances or drugs abuse, craniocerebral injury or stroke were excluded.

All the participants in both study group and control group were Han Chinese with basic abilities in reading, writing, comprehension and verbal communication. This study was approved by the Ethics Committee of No. 904th Hospital of Joint Logistic Support Force (approval number: KLS2021-11-08). Informed consent was obtained from all participants and their legal guardians.

### Evaluation scales

#### Positive and negative syndrome scale (PANSS) was used to assess psychotic symptoms in SCZ patients

PANSS comprised of 30 items including 7 items for positive symptom subscale (PSS), 7 items for negative symptom subscale (NSS), 16 items for general pathological symptom subscale (GPSS).

#### Childhood chronic stress questionaire (CCSQ) consisted of 60 items which were assigned into 3 factors of peer bullying (PB), abuse and neglect (AN) and adverse life events (ALE)

The higher the score, the more severe the chronic stress during childhood. CCSQ was firstly developed by Hu in Chinese version with Cronbach’s α of 0.946 and subscale reliabilities of 0.868, 0.835, 0.716, respectively^20^.

### Real-time quantitative reverse transcription PCR (qRT–PCR) for lncRNA expression

Based on the previous literature^16^, NONHSAT089447, NONHSAT021545, NONHSAT041499 in PBMCs were selected for further validation using qRT-PCR from 200 SCZ patients and 200 healthy controls. Total RNAs was isolated from PBMCs using TRIzol reagent for quantitative detection of lncRNAs. Complementary DNA was synthesized using the Reverse Transcription TaqMan RNA Reverse Transcription Kit. Each RT reaction included 10 μL of total RNA 3.0 μL TaqMan MicroRNA Assay, 4.16 μL nuclease free water, 0.19 μL RNase inhibitor 1 μL Multiscribe Reverse Transcriptase, 0.15 μL dNTP, 1.5 μL 10×RT buffer in a total volume of 15 μL. Reactions were performed under the following conditions: 30 min at 16 °C, 30 min at 42 °C, 5 min at 85 °C, and 10 min at 4 °C. Each sample was processed in duplicate for analysis and qRT-PCR was performed using an Applied Biosystems 7900HT Real-Time PCR System. Data were collected using SDS 2.3 software. Data were collected using the SDS 2.3 Software and DataAssist v. 3.0. The expression levels of lncRNAs were calculated using the 2^-ΔΔCt^ method after normalization to β-actin. This process was performed according to a standard detection program for lncRNA expression in PBMCs.

### Cortisol detection

A fasting blood sample (≈ 5 mL) was collected from the antecubital vein using EDTA-K tubes from 7:30 to 8:30 a.m. The blood samples were then centrifuged to collect serum at a temperature of 4 °C with a speed of 1500 rpm for 10 min and then the centrifuged serum was stored at –25 °C in a medical freezer. The serum cortisol level was detected using cortisol ELISA kits purchased from Shanghai Mingfeng Biology, and the detecting process was carried out strictly in accordance with operating manual provided by kit manufacturer.

### Statistical analysis

The ΔCt value, calculated by the threshold cycle difference (Ct) between lncRNA and β-actin (ΔCt=Ct_lncRNA_-Ct_β-Actin_), was used as the indicator of relative expression level of lncRNA, the higher ΔCt value suggested lncRNA were downregulated^16^. The between-group differences of chronic childhood stress and cortisol level were compared using the independent sample *t* test. The differences of lncRNA expression levels between the SCZ group and the control group were analyzed using Mann-Whitney U-test, receiver operating characteristic (ROC) curve was used to determine sensitivity and specificity of NONHSAT089447, NONHSAT021545, NONHSAT041499 in differentiating SCZ severity, and Logistic regression, with internal validation using Hosmer-Lemeshow goodness-of-fit test, was employed to acquire a predicted probability for the following combined ROC (*χ*^2^ = 9.041, *P* = 0.339 > 0.05). The relationship between chronic stress in childhood, lncRNA, cortisol, and psychotic symptoms in SCZ patients was determined by Pearson’s correlation analysis, Spearman’s rank correlation test, Hierarchical regression analysis. Collinearity diagnostics for regression analysis showed all Tolerances were more than 0.1, and Kolmogorov-Smirnov test verified the data of dependent variables conformed to normal distribution(*P* > 0.05). A *P* value < 0.05 was considered statistically significant.

### Ethics approval and consent to participate

The authors declare that all experiments on human subjects were conducted in accordance with the Declaration of Helsinki and approved by the Ethics Committee of No. 904th Hospital of Joint Logistic Support Force (approval number: KLS2021-11-08). Informed consent was obtained from all participants and their legal guardians.

## Discussion

Due to the high disability and recurrence rate and low cure rate, SCZ has become the main source of medical expense and family care burdens^23,24^. Therefore, the exploration for etiology and prevention strategies is of great value for improving the SCZ prognosis.

The interaction between genetic susceptibility and environmental risk factors is known as fundamental pathological mechanism that induces SCZ. This present study firstly found scores of CCSQ, PB, AN, ALE, and serum cortisol level, higher than their healthy counterparts, were positively related to scores of PANSS, PSS, NSS, GPSS in SCZ group. These forementioned results possibly indicated psychopathological symptoms of SCZ patients associated with hyperactivated stress response, chronic stress exposure in childhood. Previous studies emphasized the crucial role of chronic childhood stress in personality development, it was believed that individuals with maladaptive personality traits, such as insecure attachment, low self-esteem, and alexithymia, originated from chronic childhood stress were more prone to experience social withdrawal, social rejection, peer bullying, and social isolation which were deemed to be predictors of SCZ^25–28^. Multiple neurophysiological processes associated with exposure to chronic childhood stress are involved in the SCZ pathogenesis, several recent studies showed brain hyperactivation to present environment stimuli shaped by chronic childhood stress may in turn increased cortisol release via activating hypothalamic-pituitary-adrenal axis, impaired memory and executive control through hippocampus-prefrontal cortex interaction and amygdala-prefrontal cortex circuit, also caused hypervigilance, dissociation relating to dysfunction of serotonin, dopamine, glutamate and their regulatory pathways^29–32^, and these complex pathological changes may eventually induce SCZ.

lncRNAs, known as gene expression regulators for their function in chromosomal rearrangement and gene silencing, employed their effect in a wide variety of transcriptional and post-transcriptional regulatory mechanisms interacting with chromatin modifiers, transcription factors, and other regulatory molecules. During brain development, dysfunction of specific lncRNAs involved in neurogenesis, neuronal differentiation, neuronal survival, and synaptogenesis have proved to be implicated with disrupted neuronal connectivity, impaired synaptic plasticity, and aberrant gene expression pattern^33,34^. The verified results in this study, lowered ΔCT values of lncRNAs (NONHSAT089447, NONHSAT021545) negatively correlated with scores of PANSS, PSS, NSS, GPSS, was partially consistent with previous literature report^16^, and combined ROC curve suggested the AUC of NONHSAT089447, NONHSAT021545, NONHSAT041499 efficiently differentiating the SCZ severity with sensitivity and specificity of being 0.650, 0.880, respectively. The results of this study indicated the enriched lncRNAs with expression profiles of temporal-spatial specificity contributed to the pathological process of SCZ and efficiently differentiating the SCZ severity. In one previous study, Human neuroblastoma cell lines (SK-N-SH) were cultured and treated with olanzapine, olanzapine was proved to inhibit the expression of NONHSAT089447, while knockdown of NONHSAT089447 by siRNA decreased expression of dopamine D3/D5 receptors, and overexpression of NONHSAT089447 significantly upregulated expression of dopamine D3/D5 receptors^35^, SCZ was primarily associated with dopamine dysfunction and therapeutics for SCZ had been developed were targeting at dopamine pathway in central nervous system. Some other studies reported transcription factors (TFs), as proteins that control gene expression by binding to specific DNA sequences, are essential for cell development, differentiation, and homeostasis. Neuronal activity, including sensory-evoked and spontaneous firing, regulates the expression of a subset of genes known as activity-dependent genes, A key issue in this process is the activation and accumulation of TFs, which bind to cis-elements at specific enhancers and promoters, ultimately driving RNA synthesis through transcription machinery^36,37^. One bioinformatics analysis evaluated the contribution of lncRNAs(NONHSAT089447, NONHSAT021545, NONHSAT041499) and relevant transcription factors using interaction map with node frequency, the node frequencies of NONHSAT021545 and NONHSAT089447 were 254 and 249, respectively, which were much higher than that of NONHSAT041499 with a node frequency of 26, the bioinformatics results suggested NONHSAT021545 and NONHSAT089447 regulated much more target genes than NONHSAT041499. Among the TFs, TAF1, EBF1 and USF1 with highest node frequencies were proved to be associated with SCZ^38–40^. It could be considered in summary that aberrant expressed lncRNAs would trigger SCZ pathogenesis via regulatory mechanisms of neurotransmitter and TFs.

Epigenetics mainly explored the mediators or bridges between relevance of SCZ susceptibility genes and environmental stimuli, among which lncRNA, as an epigenetic regulator, has been brought to the forefront in field of mental health promotion and neuropsychiatric disease treatment^41,42^. The mediating effect analysis in this study revealed lncRNAs (NONHSAT089447, NONHSAT021545) played as the bridges linking chronic childhood stress and psychopathological symptoms in SCZ patients which could respectively account for 54.83% and 41.53% of total effect. The conclusion from this present study, chronic childhood stress could also indirectly induce pathological process and onset of SCZ by modulating expression profile and function of lncRNA, was consistent with previous theoretical hypothesis^43,44^. Nevertheless, the knowledge about how environmental stress changed the expression pattern and function of lncRNA was still limited^45^, some researches seemed to provide some explorative orientations, one study reported a 3-week enriched environment intervention on rat model of depression induced by chronic unpredictable mild stress could ameliorate depression-like behavior, cognitive impairment and elevate neuronal dendritic spine density and KLF2 level, but reduced miR-92a-3p level in hippocampal tissues, while another study suggested miR-92a-3p would upregulated in patients with depression and anxiety relating to drug abuse^46,47^. Notably, recent studies reported ncRNAs (miRNAs, lncRNAs, circRNAs) may form regulatory networks for performing their functions, miRNAs acted as molecular scaffolds, decoys, guides, or sponges for lncRNAs could emerge as crucial regulators in diverse biological processes including chromatin remodeling, transcriptional regulation, and epigenetic control, gene expression^48,49^. Transcriptional and translational effects which ameliorated experience-dependent cellular plasticity and cognitive performance could be enhanced in animals housed under enriched environment conditions; Enriched environment was shown to induce nerve repair by restoring functional activities through morphological, cellular, and molecular adaptations in the brain that have clinical relevance, delaying the onset and progression of a wide variety of symptoms in SCZ patients^50^, therefore, altered expression pattern and function of miRNA may regulate the improvement process of SCZ symptoms based on enriched environment and conversely changed lncRNA expression profile. It could be inferred that chronic childhood stress and hyperarousal of stress response system caused by ELS would regulate expression level of lncRNA by altered miRNA through circRNA/lncRNA-miRNA-mRNA network. In addition, some other studies suggested ncRNA (miRNA and lncRNA) would be aberrantly expressed under the condition of inflammatory cytokines relating to SCZ pathogenesis. One recent study found peripheral inflammation markers (IL-1β, IL-6, high sensitivity C-reactive protein and/or neutrophil to lymphocyte ratio) were elevated and canakinumab treatment for 8 weeks could quickly reduce high sensitivity C-reactive protein with ameliorated positive symptom severity in SCZ patients. Environmental stimuli and stress responses were usually the triggering factors for human inflammatory responses^51–55^. Therefore, inflammatory mechanism maybe another physiological bases for lncRNA expression patterns in SCZ patients under stress conditions.

**Fig. 3:**
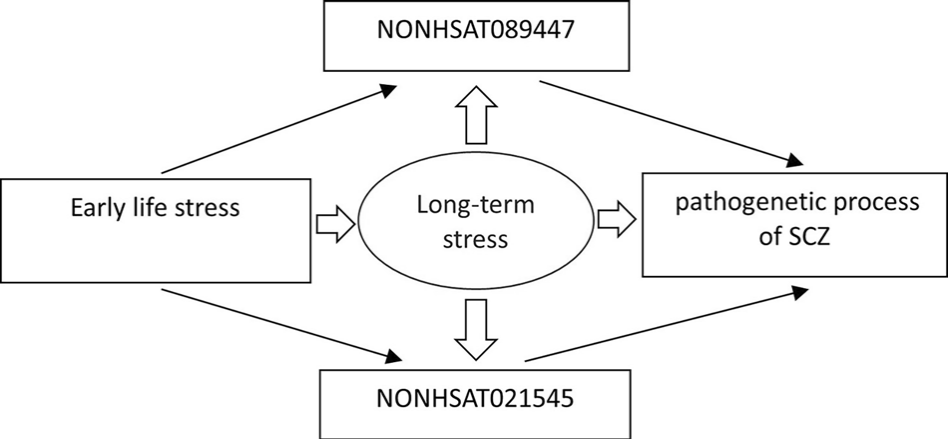
A flow diagram for mediating effects of NONHSAT089447 and NONHSAT021545between chronic childhood stress and SCZ.

This study has made contributions to the fields of basic research and clinical treatment for SCZ in the following aspects. Firstly, it confirmed chronic childhood stress, lncRNA expression levels, and cortisol were predictors of psychopathological symptoms in SCZ patients, providing new evidence based on human patients for the neurodevelopmental model of SCZ^56,57^; Secondly, the results of ROC curve for differentiating value of lncRNA on SCZ severity in this study indicated NONHSAT089447, NONHSAT021545 may serve as potential biomarkers for therapeutic response of SCZ featuring with complex symptoms and disease-course fluctuations. Based on multicenter, double-blind verification, a SCZ-specific detection kit for lncRNA level, which could provide reference indicator for SCZ patients’ health condition assessment and treatment plan adjustment, may be developed and applied in clinical practice; Thirdly, verification of the mediating role of lncRNAs (NONHSAT089447, NONHSAT021545) between chronic childhood stress and psychopathological symptoms in SCZ patient could provide new knowledge about epigenetic mechanism of SCZ. A new model for SCZ in which exposure to chronic childhood stress could induce pathological process via mediating effects of NONHSAT089447 and NONHSAT021545 could established based on this study (Fig.3). Lastly, Given the link between environmental stimuli, genetic, epigenetic alterations and reversible nature of epigenetic mechanisms, the SCZ susceptibility genes would be inhibited by altered expression pattern and function of lncRNAs relevant to the environment improvement^50,58,59^, therefore, strengthening ELS management for vulnerable population may be a crucial link in the SCZ prevention.

Some limitations and future directions needed to be noted, current researches still had limited knowledge about the molecular basis by which environmental stress affected lncRNA expression and the specific function of NONHSAT089447, NONHSAT021545 in the SCZ pathogenesis and regulatory pathways, hypotheses primarily proposed in this paper about the miRNA-lncRNA regulatory network and inflammatory mechanism were necessary to be validated in the future. As a cross-sectional study, we could not draw a causal conclusion, and the data of chronic childhood stress was assessed via psychological scale, which may limit result reliability due to recall bias, animal model and longitudinal studies on the association between chronic childhood stress, lncRNAs, and SCZ may enhance the validity and reliability findings in this study. Some multi-center and double-blind verifications for the value of SCZ-specific lncRNA expression pattern in SCZ diagnosis, severity assessment seem to be a problem that needs to be solved in future research and clinical practices. Furthermore, selection bias for participants enrollment needs to be noted in the future due to confounding effects of some possible variables (e.g., smoking, BMI, and inflammation markers).

## Conclusion

This study found that chronic childhood stress, NONHSAT089447, NONHSAT021545, and cortisol are predictors of schizophrenia and chronic childhood stress can also induce the SCZ pathogenesis through the mediating effects of lncRNAs (NONHSAT089447 and NONHSAT021545). The results in this study provide new evidence for the neurodevelopmental model and epigenetic hypothesis and offer new directions for prevention and rehabilitation of SCZ.

## Data Availability

The data supporting the results of this study are available from the corresponding author upon reasonable request.
